# Synopsis of Essay on Classification of Skin Diseases

**Published:** 1873-12

**Authors:** J. W. Southworth

**Affiliations:** Toledo, Ohio


ART. III.—*Synopsis of Essay on Classification of Skin Diseases.* By J. W. SOUTHWORTH, M. D., Toledo, Ohio.

The dermatologist cannot hope to arrange a perfectly scientific, much less a *systematic* practical classification the affections under consideration, founded *solely* on either Etiology, Pathology, Clinology, or Morbid Anatomy; unless these subjects are more fully elucidated. The skin is simply one of the complex organs of the body, with which it participates more or less in the various changes, laws and sympathies (both in health and disease) by reason of its intimate and extensive anatomical relations, as well as by its histological analogy. The physiology of the one, then, must become the physiology of the other, and consequently the pathology of the one is equally certain to be that of the other.

Why then may we not say the same of nosology? Most assuredly we can and should do so, as far as possible, without compromising its practical utility.

The so-called pathological classification of Hebra is conceded to be a decided step in advance, but it serves the morbid anatomist much more than it does the practitioner. Though aiming to make it correspond in plan to the pathology of the general system, he finds himself obliged to bring etiology, morbid anatomy, &c., to his aid, and we get mainly an anatomico-pathological classification. In his class "Exudata" or inflammatory exudates, is included, not only the majority of skin diseases, but also those widely separated in their essential pathology, viz.: Acne Vulgaris and malignant pustule; or again eczema and small pox; grouping them in this class, *solely* because they all present in common *an inflammatory exudate.*

Prof. Neuman has indeed simplified it in his hand-book of skin diseases, and made it appear less mystifying to the student; though it must be confessed that his arrangement of the syphilitic and lupous affections, in the same class with vascular and adipose tumors, does not add much to the fullest appreciation of the subject, even if they are all styled "neoplasms" or newformations.

The arrangement which brings together in classes those affections having a similarity of known cause, in others those having the greatest affinity in pathological nature, in others those having most nearly corresponding clinical characteristics would be most highly advantageous; all being subdivided into *groups* having the closest resemblance in symptoms and also requiring the same principles of treatment.

Alibert of France essayed some such practical classification in 1810, but on account of its defective grouping, (e. g. such as putting boils and burns in the same class with erysipelas and erythema in his "eczematous" or eruptive class: also the inclusion of scabies and prurigo together in his "scabious" class,) his "tree of the dermatoses" did not long survive.

Later, we find Hardy following in his footsteps and adding greatly to its value by merging some of the affections into other groups where they more naturally belonged, making ten classes as follows: Maculae (deformities) local inflammations, porasitic affections, eruptive fevers, symptomatic eruptions, dartres, scrofulides, syphilides, cancers, and exatic affections.

Another author of the French school M. Devergie, though dissatisfied with preceding efforts, admitted his inability to improve upon them, and endeavored to frame one which should "have the appearance of logical order," guided as he states by the following leading ideas, viz: 1st. Analogy of Cause. 2d. Morbid Forms. 3d. Morbid Products. 4th. Climate Origin. From these headings fourteen classes were evolved, including the appendages of the skin. Only two of the fourteen classes had definitive titles to wit: Syphilides, and diseases of the nails, the rest were distinguished from each other by numerals.

But we must turn our eyes from sunny France to England's sombre shores if we would see the master hand in the production of a most admirable nosologic arrangement, styled by its distinguished author "Clinical Classification." Prof. Wilson's arrangement is eminently practical but there is too much of it, too many classes to best subserve the highly important aim of simplicity, or ease of comprehending the *inseparable relations* of cutaneous pathology and therapeutics to that of the general system. It leads the student to think there is such a multiplicity of distinct and peculiar morbid processes relating to the skin, that he shrinks from what seems to be a most difficult problem, and consoles himself with the fact that but few practitioners attain a satisfactory proficiency in the management of this class of diseases.

The Clinical Classification of Wilson, is as follows: "(1) Eczematous, (2) erythematous, (3) bullous, (4) furunculous, (5) nervous, (6) vascular, (7) hæmic, (8) developmental and nutritive, (9) hypertrophic and atrophic, (10) alphous, (11) strumous, (12) cancerous, (13) zymotic, (14) syphilitic, (15) leprous, (16) prigmentary, (17) phytodermic, (18) ungual, (19) pilary, (20) sebiperous, (21) sudoriperous, and (22) traumatic affections."

It is only with the members of the 'eczematous' and 'alphous' classes that we question the propriety of so arranging them; as in the former he includes scabies with eczema, lichen and ptyriasis, etc., because he considers the dermatitis traumatically excited by the acarus, *an eczema* produced by such special cause. We might with as much propriety or force of argument maintain that the wheals arising from the attacks of musquitos was nettle rash! Scabies must therefore according to both reason and analogy, be placed among the parasitic affections with its congeners, the acaro-dermata.

The 'Alphous' class, consisting of one lone member Alphos, or lepra vulgaris, (being so far, as yet, determined, but a chronic diathetic inflammatory hyperplasia) has no sufficient points of difference to entitle it to a separation from all the rest of cutaneous diseases; so we have placed it in our class of diathetic inflammations. In this we are in accord with Hardy, who uses the term 'Dartres' as synonymous with the eczematous class of Wilson—which latter forms one of the clinical groups in our own class above referred to.

It is not however without due reverence for so high an authority that I present a far simpler arrangement which shall embrace the essential elements of clinical classification, having for its object the same practical aim. A much larger number of diseases are thus shown to be pathologically and etiologically allied to each other. Also the terms used in designating most of the classes "clearly embody a precise idea, as well as express. The *essential nature of the morbid action pertaining to each in common;* which latter cannot but lead to appropriate and rational treatment. In other classes, their well recognized similarity of causative relations are embodied in the terms applied to each, thus more definitely contributing to a practical end, whilst in sub-grouping the different affections in each class, the clinical, etiological, or pathological relations are as precisely and distinctively retained and expressed, as if no primary or general classification had been made.

This I consider a most valuable feature of the plan as it establishes more clearly the common affinities or relations of the different members of the same group.

The following schedule will show at a glance the advantages claimed:

CLASS I.—DIATHETIC INFLAMMATIONS—NON-CONTAGIOUS.

*Clinical Groups*—Erythematous: Erythema, urticaria, roseola and acrodynia. Eczematous: Eczema,* lichen, pityriasis and lepra vulgaris, or alphos. Bullous: Herpes, hydroa and pemphigus. Scrofulous: The scrofulo-dermata. Lupous: The lupo-dermata. Phlegmonous: Ecthyma, furuncle, carbuncle, and abscess, or phlegmono-dermata.

CLASS II.—TOXÆMIC INFLAMMATIONS—CONTAGIOUS OR INFECTIOUS.

*Clinical Groups*—Exanthematous: Scarlatina, rubeola and rubella. Variolous: Variola, varioloid, vaccinia, and varicella. Septic: Erythema, erysipelas and malignant pustule. Farcinomic: The farcino-dermata. Syphilitic: The syphilo-dermata.

CLASS III.—ATROPHY—OF GENERAL OR SPECIAL TISSUES.

*Clinical Groups*—Senile, nervous, congenital, etc.

CLASS IV.—HYPERTROPHY—OF GENERAL OR SPECIAL TISSUES.

*Clinical Groups*—Mechanical, congenital, inflammatory, etc.

CLASS V.—HETEROTROPHY—OF GENERAL OR SPECIAL TISSUES.

*Clinical Groups*—Cancerous and tubercular.

CLASS VI.—NEUROTIC AFFECTIONS.

*Clinical Groups*—Spinal and sympathetic neuroses.

CLASS VII.—GLANDULAR AFFECTIONS.

*Clinical Groups*—Sudatory and sebiperous disorders.

---

* Eczema includes impetigo, gutta rosacea and psoriasis of Wilson.

CLASS VIII.—TRAUMATIC AFFECTIONS.

*Clinical Groups*—Mechanical, thermal and chemical.

CLASS IX.—THERAPEUTIC AFFECTIONS.

*Clinical Groups*—From internal medication. From external applications.

CLASS X.—HÆMORRHAGIC AFFECTIONS.

*Clinical Groups*—Dyscrasic and Symptomatic.

CLASS XI.—ENDEMIC CONSTITUTIONAL AFFECTIONS. (Pellagra, Leprosy, Yaws, &c.)

CLASS XII.—PARASITIC AFFECTIONS.

*Clinical Groups*—Phytoform and zooform.

The above affords a marked contrast with the most recent author of the English School, (Dr. Fox) in this, that he includes under the head of "Local Inflammations," all the affections coming under or belonging to the class "Diathetic Inflammations" of my own table. In class first, "Eruptions of acute Specific Diseases." Dr. Fox has with the exception of Syphilis, almost paralleled my second class;—"Toxæmic Inflammations. Though why he should see fit to associate a disease due to a special blood poison with others which are not, such as Scrofula and Leprosy, is shown by the fact of his entitling them "Diathetic Diseases." But surely are not other affections such as Lepra vulgaris and Eczema, &c., equally entitled to be called diathetic? Besides there is also the impropriety of associating contagious diseases with those which are not. Leprosy, another member of his "Diathetic" class I have thought best to include in a separate group, viz: "Endemic Constitutional Affections," in which the cutaneous lesions are symptomatic of the morbid processes going on in general system.

With the remaining classes of Dr. Fox I see no objection. In fact his arrangement is superior to most of his predecessors, in simplicity and practical utility. It is only with the insufficiency of the terms used to designate some classes, and the inconsistency of making a separate class of diathetic diseases, that I would feel justified in criticising as above; and not wishing to do him injustice, will append his classification:—Classification of Tilbury Fox.

CLASS I.—ERUPTIONS OF ACUTE SPECIFIC DISEASES.

CLASS II.—LOCAL INFLAMMATIONS.

*Clinical Groups*—Erythematous, Catarrhal, Plastic, Suppurative, Bullous and Squamous.

CLASS III.—DIATHETIC DISEASES.

*Clinical Groups.*—Scrofulous, Syphilitic and Leprous.

CLASS IV.—HYPERTROPHIES AND ATROPHIES.

*Clinical Groups*—Epithetial, Fibrous, Papillary, Vascular: Senile atrophy and developmental diseases.

CLASS V.—NEOPLASMS. (Cancer, lupus, rodent ulcer.)

CLASS VI.—HEMORRHAGIC AFFECTIONS.

CLASS VII.—NEUROTIC AFFECTIONS.

CLASS VIII.—PIGMENTARY AFFECTIONS.

CLASS IX.—GLANDULAR AFFECTIONS AND APPENDAGES.

*Clinical Groups*—Affections of sebiperous glands and sudoriferous glands, hair and nails.

CLASS X.—PARASITIC AFFECTIONS.

*Clinical Groups*—Phytoform and zooform.

